# The role of transoral robotic surgery, transoral laser microsurgery, and lingual tonsillectomy in the identification of head and neck squamous cell carcinoma of unknown primary origin: a systematic review

**DOI:** 10.1186/s40463-016-0142-6

**Published:** 2016-05-04

**Authors:** Terence S. Fu, Andrew Foreman, David P. Goldstein, John R. de Almeida

**Affiliations:** Faculty of Medicine, University of Toronto, Toronto, ON Canada; Department of Otolaryngology – Head and Neck Surgery, University of Toronto, Toronto, ON Canada

**Keywords:** Unknown primary, TORS, TLM, Lingual tonsillectomy, Cervical metastases

## Abstract

**Background:**

Squamous cell carcinoma of the head and neck can present as a cervical metastasis from an unknown primary site. Recently, transoral robotic surgery (TORS) and transoral laser microsurgery (TLM) have been incorporated in the workup of unknown primary tumors.

**Methods:**

We searched MEDLINE, EMBASE, Cochrane, and CINAHL from inception to June 2015 for all English-language studies that utilized TORS, TLM, or lingual tonsillectomy in the approach to an unknown primary.

**Results:**

Of 217 identified studies, eight were reviewed. TORS/TLM identified the primary tumor in 111/139 (80 %) patients overall, and 36/54 (67 %) patients with no remarkable findings following physical exam, radiologic imaging, and panendoscopy with directed biopsies. Lingual tonsillectomy identified the primary tumor in 18/25 (72 %) patients with no findings. Hemorrhage (5 %) was the most common perioperative complication.

**Conclusion:**

Lingual tonsillectomy using new approaches such as TORS/TLM may improve the identification of occult primary tumors.

## Background

Cervical metastases from an unknown primary tumor site account for 2 to 5 % of all squamous cell carcinoma of the head and neck [1, 2]. Identification of the primary site may have an impact on disease control and survival, in addition to potentially minimizing treatment-related toxicity from large volume head and neck mucosal irradiation [2–7].

The standard workup of an unknown primary consists of a history, physical examination with flexible endoscopy, and diagnostic imaging such as computed tomography (CT) and/or magnetic resonance imaging (MRI). Positron-emission tomography (PET), alone or fused with CT images (PET-CT), may improve the diagnostic sensitivity when traditional imaging modalities fail to localize a primary tumor [1, 4, 8, 9]. When the primary tumor remains elusive despite these modalities, examination under anesthesia with panendoscopy and directed biopsies of the nasopharynx, hypopharynx, and oropharynx has been the traditional approach. The definition of an unknown primary is neither absolute nor static; a primary tumor site identified by any diagnostic modality is, by definition, no longer an unknown primary. Despite this extensive workup, however, over 50 % of primary tumors remain undiscovered [1, 3, 10, 11].

In the absence of a visible or palpable lesion, a palatine tonsillectomy may improve the diagnostic yield of an occult primary tumor compared to deep tonsil biopsies [12–14], as many occult primaries maybe hidden deep in tonsillar crypts. [12] Given that 80–90 % of occult primary tumors are eventually localized in the palatine tonsil and tongue base, palatine and lingual tonsillectomies have been recognized as important additions to the diagnostic workup of an unknown primary [1, 5, 10].

Recently, Transoral Laser Microsurgery (TLM) and Transoral Robotic Surgery (TORS) have emerged as effective modalities to aid in the identification and treatment of an unknown primary tumor. These techniques provide enhanced visualization and maneuverability, allowing for a complete resection of the entire tongue base mucosa and lingual tonsils, a procedure which is challenging to perform using traditional instrumentation and visualization [15, 16]. Recent case series of occult primary tumors have reported high rates of detection ranging from 86 to 94 % using TLM [16, 17], and 72 to 90 % using TORS [15, 18, 19]. However, these studies contain small, heterogeneous patient populations with variable preoperative investigations and findings, and thus cannot be directly compared.

The present study aims to conduct a systematic review of the literature to determine the incremental benefit of lingual tonsillectomy using TORS/TLM in localizing the primary tumor site of regionally metastatic head and neck squamous cell carcinoma of unknown origin.

## Methods

### Search strategy

A systematic review of published reports on TORS or TLM for the workup of CUP was performed. MEDLINE, EMBASE, Cochrane Central Register, and CINAHL were searched from inception to June 2015 for all relevant English-language studies. Medical Subject Headings and keywords specifying histopathology (e.g. squamous cell carcinoma), location (e.g. head and neck, cervical metastases), unknown primary, and diagnostic approach (e.g. TORS, TLM, or lingual tonsillectomy) were used to identify studies. Bibliographies of all included studies were also searched for relevant articles.

### Selection criteria

**Fig. 1:**
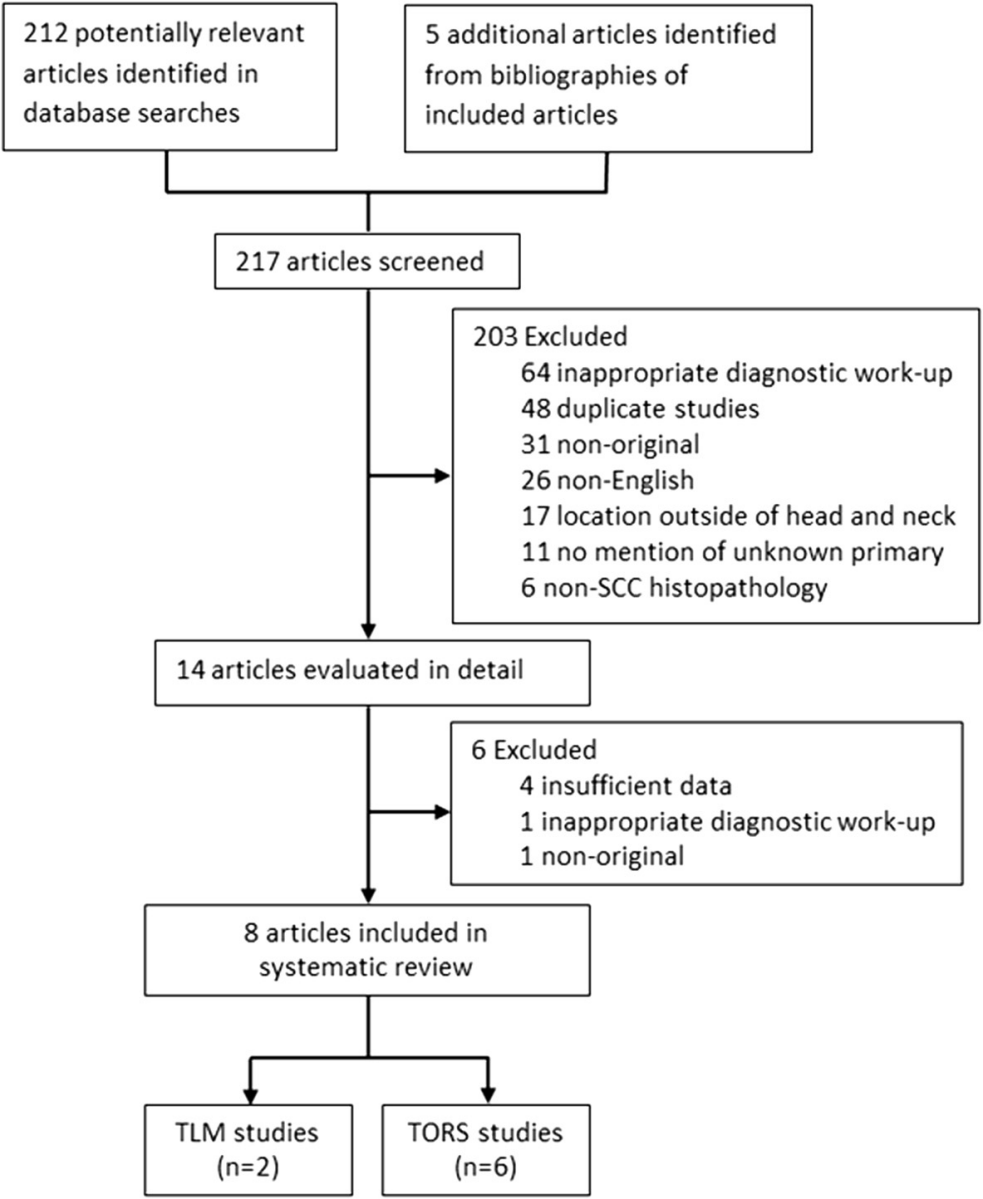
Selection of studies for systematic review

Two reviewers (T.F. & A.F.) independently screened all identified studies by title and abstract for further full text review, and then independently reviewed these studies for eligibility (Fig. 1). Studies were included if they used TORS, TLM, or lingual tonsillectomy via TORS/TLM in the diagnostic approach to head and neck squamous cell carcinoma of unknown primary origin. Non-English and non-original studies (i.e. reviews) were excluded. When multiple studies were published by a single institution, only the most recent study was included to avoid inclusion of the same patients more than once in the review. Disagreements were resolved by consensus.

### Data extraction & statistical analysis

Data were extracted in duplicate by two reviewers (T.F. & A.F.). The primary outcome was the identification rate of an unknown primary site using TORS, TLM, or lingual tonsillectomy performed using TORS/TLM. Information on study design, patient and tumor characteristics, diagnostic workup, margin status, and perioperative complications was also extracted. Subgroup analysis of identification rates were performed based on the presence or absence of positive findings on preoperative investigations including [1]: physical examination (PE) [2], diagnostic imaging (DI) consisting of computed tomography or magnetic resonance imaging (CT or MRI) [3], positron emission tomography-computed tomography (PET-CT) [4], a combination of PE/DI/PET-CT, and [5] examination under anesthesia (EUA) with directed biopsies of the nasopharynx, hypopharynx, tonsil, and base of tongue. Data were aggregated using Microsoft Excel 2010 (Microsoft Corp., Redmond, Washington), and all statistical analyses were conducted using SPSS version 21.0 (SPSS Inc., Chicago, Illinois).

## Results

### Study selection

The literature search identified a total of 217 articles (Fig. 1). Excluded studies included those that did not use TORS, TLM, or lingual tonsillectomy (64), duplicates (48), non-original studies (31), non-English studies (26), studies of non-head and neck neoplasms (17), studies without mention of unknown primary (11), and those reporting on non-SCC histopathology (6). Of the 14 remaining studies, three were follow-up studies [20–22] from the same institution, one study [23] was excluded due to insufficient data, one study [24] did not use TORS or TLM in the diagnostic workup, and one study [25] was a review paper. Inter-rater agreement for study inclusion was excellent (*κ* = 0.92).

Eight studies containing a total of 139 patients met the final inclusion criteria [15–19, 26–28]. Of these eight studies, six studies [15, 18, 19, 26–28] reported outcomes for 85 patients undergoing TORS for workup of an unknown primary, and two studies [16, 17] reported outcomes for 54 patients undergoing TLM.

### Study characteristics

Characteristics of the eight included studies are summarized in Table 1. Included studies were case series or case reports published between 2011 and 2014. All were single-institution studies aside from one study [15] which pooled data from six institutions.

**Table 1:** Summary of studies included in systematic review

Authors	Year	Institution	No. Pts (*N* = 139)
Abuzeid et al. [26]	2011	University of Michigan	1
Blanco et al. [28]	2013	Johns Hopkins School of Medicine	4
Durmus et al. [18]	2013	Ohio State University Wexner Medical Center	22
Karni et al. [16]	2011	Washington University School of Medicine	18
Mehta et al. [19]	2013	University of Pittsburgh Medical Center	10
Mourad et al. [27]	2013	Albert Einstein College of Medicine	1
Nagel et al. [17]	2014	Mayo Clinic Arizona	36
Patel et al. [15]	2013	University of Washington Medical Center, University of Texas MD Anderson Cancer Center, University of Alabama-Birmingham Hospital, University of Texas Medical School at Houston, Johns Hopkins Hospital, Oregon Health Sciences University	47

Patient characteristics are summarized in Table 2. The mean age of patients undergoing TORS or TLM was 57.3 years (standard deviation [SD] 2.1, range 44–78 years). Patients were predominantly male (88 %), and the majority (82 %) of the 65 patients with a reported p16 status were positive [15, 18, 19, 26, 27]. Of the 94 patients with known nodal status, 19 (20 %) were N1, 62 (66 %) were N2, and 13 (14 %) were N3. The mean diameter of identified primary tumors was 1.15 cm (SD 0.79 cm, range 0.2 to 3.0 cm). Of 71 patients with known margin status, 44 (62 %) had negative margins [15, 18, 26, 27].

**Table 2:** Characteristics of patients from included studies

Characteristic	No. Pts (%) (*N* = 139)
Age, mean (SD)	57.3 (2.1)
Sex	
Female	16 (12 %)
Male	119 (88 %)
na	4
HPV	
+	53 (82 %)
-	12 (18 %)
na	74
Nodal status	
N1	19 (20 %)
N2	62 (66 %)
N3	13 (14 %)
na	45
Size, mean cm (SD)	1.15 (79 %)
Negative Margins	44 (62 %)

### Diagnostic workup of unknown primary

The diagnostic workup for an unknown primary was highly variable between institutions as shown in Table 3. PE findings were suspicious for a primary tumor in 24 of 135 (18 %) patients. Nine of 89 (10 %) patients had suspicious findings on DI, and 17 of 39 (44 %) patients had findings on PET-CT scan. Of the 78 patients undergoing a full diagnostic workup including PE/DI/PET-CT, 43 (55 %) had suspicious findings. EUA with biopsies of the nasopharynx, hypopharynx, and oropharynx revealed remarkable findings in 12 of 52 (23 %) patients. All 12 patients with findings on EUA received a lingual tonsillectomy using TORS.

**Table 3:** Diagnostic workup and proportion of patients with suspicious findings (*n* = 139)

Investigation	Proportion of patients with suspicious findings	Proportion of patients without suspicious findings	No. Patients with Missing Data
Physical Exam	24/135 (18 %)	111/135 (82 %)	4
DI (CT/MRI)	9/89 (10 %)	80/89 (90 %)	41
PET-CT	17/39 (44 %)	22/39 (56 %)	100
PE/DI/PET-CT	43/78 (55 %)	35/78 (45 %)	61
EUA with biopsy	12/52 (23 %)	40/52 (77 %)	87

*Abbreviations*: *DI* diagnostic imaging, *CT* computed tomography, *MRI* magnetic resonance imaging, *PE* physical examination, *PET* positron emission tomography, *EUA* panendoscopic examination under anesthesia

A total of 108 of 139 patients (78 %) underwent lingual tonsillectomy by TORS or TLM. Of the 90 patients with available information, 36 (40 %) had ipsilateral lingual tonsillectomy and 54 (60 %) had bilateral lingual tonsillectomy. Three studies [17, 19, 26] explicitly described the procedure for performing lingual tonsillectomy. The procedure was generally consistent across all three institutions and involved complete resection of the lingual tonsil from the midline of the tongue to the lateral pharyngeal wall, and from the circumvallate papillae to the vallecula, using the muscular layer as the deep plane of dissection.

A total of 70 of 103 (68 %) patients underwent palatine tonsillectomy by TORS or TLM. Palatine tonsillectomy was either not performed or not reported in the remainder of the patients (36 of 139) for the following reasons: (i) 20 patients in one series [15] did not undergo palatine tonsillectomy, (ii) at least ten patients had a previous childhood tonsillectomy [18, 19, 26], and (iii) one study [17] did not report the frequency of palatine tonsillectomies in the TORS/TLM group. Among the 55 patients undergoing palatine tonsillectomy with available information, 24 (44 %) had ipsilateral tonsillectomy while 31 (56 %) had bilateral tonsillectomy.

### Identification of unknown primary using TORS/TLM

Overall, TORS/TLM successfully localized the primary tumor in 111 of 139 (80 %) patients, as shown in Table 4. An occult primary was identified in 60 of 108 (56 %) patients undergoing lingual tonsillectomy and 34 of 70 (49 %) patients undergoing palatine tonsillectomy using TORS or TLM. One patient undergoing TORS had synchronous primary tumors found in the palatine and lingual tonsils [15]. The location of the primary tumor was not specified in the remaining 18 of 111 patients with an occult tumor found on TLM [17].

**Table 4:** Overall identification rate of unknown primary with TORS/TLM

Author	Method	Proportion identified with TORS/TLM	Proportion identified with lingual tonsillectomy using TORS/TLM	Proportion identified with palatine tonsillectomy using TORS/TLM
Abuzeid et al. [26]	TORS	1/1 (100 %)	1/1 (100 %)	0/0 (0 %)^a^
Blanco et al. [28]	TORS	1/4 (25 %)	0/4 (0 %)	1/4 (25 %)
Durmus et al. [18]	TORS	17/22 (77 %)	4/14 (29 %)	13/17 (76 %)
Karni et al. [16]	TLM	17/18 (94 %)	11/18 (61 %)	6/18 (33 %)
Mehta et al. [19]	TORS	9/10 (90 %)	9/10 (90 %)	0/3 (0 %)^b^
Mourad et al. [27]	TORS	1/1 (100 %)	1/1 (100 %)	0/1 (0 %)
Nagel et al. [17]	TLM	31/36 (86 %)	13/19 (68 %)	-
Patel et al. [15]	TORS	34/47 (72 %)	21/41 (51 %)	14/27 (52 %)^c^
**Total**	**TORS/TLM**	**111/139 (80 %)**	**60/108 (56 %)**	**34/70 (49 %)**

^a^Patient had childhood tonsillectomy

^b^Seven of ten patients had childhood tonsillectomy

^c^One patient had synchronous lingual/palatine tonsil tumors

**Fig. 2:**
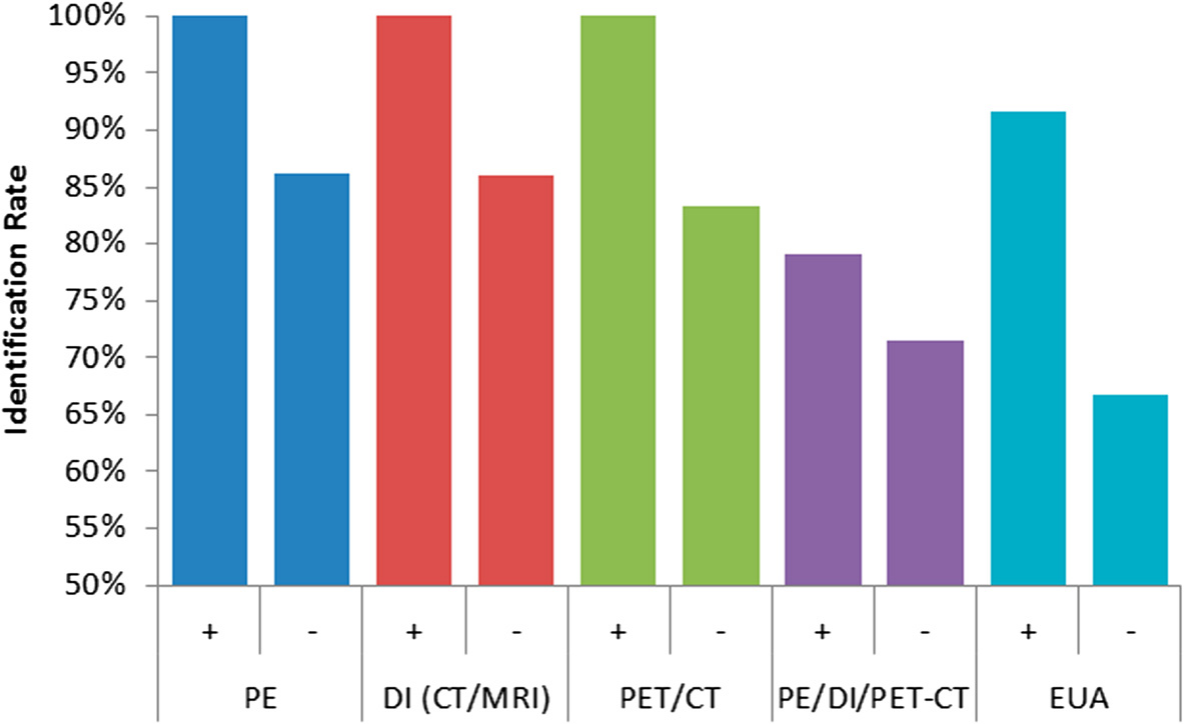
Identification of unknown primary using TORS/TLM in the presence (+) or absence (-) of other findings

Identification rates for subgroups of patients with positive or negative findings during preoperative investigations are shown in Fig. 2 and Table 5. In some studies, identification rates were reported for the entire cohort and not stratified by subgroup of patients with or without abnormal findings, thus limiting the extractable data. Only one study [26] described the identification rate in patients with positive physical exam findings. The occult primary was eventually localized in this patient (100 %) with suspicious findings on PE. In contrast, the identification rate was 86 % (75 of 87) among patients without exam findings [16–19, 27]. The primary tumor was also identified in 1 of 1 (100 %) patient with suspicious findings on DI [26], and 43 of 50 (86 %) patients without DI findings [16, 18, 19]. A primary tumor was identified in six of six (100 %) patients with remarkable findings on PET-CT [19, 26, 27], and five of six (83 %) patients without PET-CT findings [19]. TORS/TLM localized the primary tumor site in 34 of 43 (79 %) patients with remarkable findings on either PE, DI, or PET-CT [15, 18, 19, 26, 27], and 25 of 35 (71 %) patients without findings on these investigations [15, 18, 19]. In addition, a primary tumor was identified in 11 of 12 (92 %) patients with findings on EUA with directed biopsies [18, 27], but only 36 of 54 (67 %) patients without EUA findings [15, 17–19, 26]. Although a total of 34 palatine tonsil primaries were identified, the location was specified in only 13 cases [18]. Of these 13 cases, 11 (85 %) were identified in the ipsilateral tonsil, and 2 (15 %) were found in the contralateral tonsil.

**Table 5:** Identification rate of TORS/TLM in the presence of other findings

Author	Physical Exam		DI (CT/MRI)		PET/CT		PE/DI/PET-CT		EUA with biopsy	
	+	-	+	-	+	-	+	-	+	-
Abuzeid et al. [26]	1/1 (100 %)	0/0 (0 %)	1/1 (100 %)	0/0 (0 %)	1/1 (100 %)	0/0 (0 %)	1/1 (100 %)	0/0 (0 %)	0/0 (0 %)	1/1 (100 %)
Blanco et al. [28]	-	-	-	-	-	-	-	-	-	-
Durmus et al. [18]	0/0 (0 %)	17/22 (77 %)	0/0 (0 %)	17/22 (77 %)	-	-	10/11 (91 %)	7/11 (64 %)^a^	10/11 (91 %)	7/11 (64 %)
Karni et al. [16]	0/0 (0 %)	17/18 (94 %)	0/0 (0 %)	17/18 (94 %)	-	-	-	-	-	-
Mehta et al. [19]	0/0 (0 %)	9/10 (90 %)	0/0 (0 %)	9/10 (90 %)	4/4 (100 %)	5/6 (83 %)	4/4 (100 %)	5/6 (83 %)	0/0 (0 %)	9/10 (90 %)
Mourad et al. [27]	0/0 (0 %)	1/1 (100 %)	-	-	1/1 (100 %)	0/0 (0 %)	1/1 (100 %)	0/0 (0 %)	1/1 (100 %)	0/0 (0 %)
Nagel et al. [17]	0/0 (0 %)	31/36 (86 %)	-	-	-	-	-	-	-	8/14 (57 %)
Patel et al. [15]	-	-	-	-	-	-	18/26 (69 %)^b^	13/18 (72 %)	0/0 (0 %)	11/18 (61 %)^c^
**Total**	**1/1 (100 %)**	**75/87 (86 %)**	**1/1 (100 %)**	**43/50 (86 %)**	**6/6 (100 %)**	**5/6 (83 %)**	**34/43 (79 %)**	**25/35 (71 %)**	**11/12 (92 %)**	**36/54 (67 %)**

*Abbreviations*: *DI* diagnostic imaging, *CT* computed tomography, *MRI* magnetic resonance imaging, *PE* physical examination, *PET* positron emission tomography, *EUA* panendoscopic examination under anesthesia

^a^No suspicious findings on PET/CT, EUA, directed biopsies, or robotic exam

^b^Denominator was calculated as 47 total patients minus 18 patients without positive findings minus three patients who did not undergo radiographic imaging before TORS

^c^Failed deep tongue base biopsy

### Identification of unknown primary using lingual tonsillectomy

**Fig. 3:**
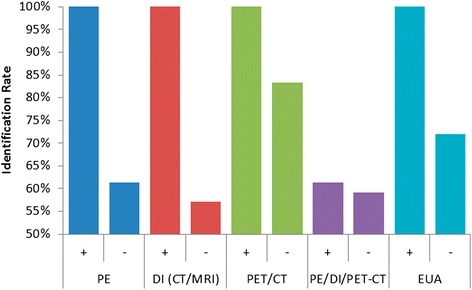
Identification of unknown primary using lingual tonsillectomy in the presence (+) or absence (-) of other findings. Abbreviations: PE, physical examination; DI, diagnostic imaging; CT, computed tomography; MRI, magnetic resonance imaging; PET, position emitted tomography; EUA, examination under anesthesia with directed biopsy

Similarly, identification rates were recorded for a subgroup of patients who underwent lingual tonsillectomy performed using TORS or TLM (Fig. 3 and Table 6). A primary tumor site was localized in 1 of 1 (100 %) patient with suspicious PE findings [26], and 38 of 62 (61 %) of patients without suspicious findings [16–19, 27]. The primary tumor was also identified in the same 1 of 1 (100 %) patient who also had findings on DI [26], and 24 of 42 (57 %) patients without findings on DI [16, 18, 19]. Lingual tonsillectomy identified the primary tumor in six of six (100 %) patients with remarkable findings on PET-CT [19, 26, 27], and five of six (83 %) without PET-CT findings [19]. Of the 31 patients with suspicious findings on either PE, DI, or PET-CT, 19 (61 %) were successfully identified [15, 19, 26, 27], while 13 of 22 (59 %) patients without findings were identified [15, 19]. A primary tumor was identified in 1 of 1 (100 %) patient with positive findings on EUA with biopsy [27], and 18 of 25 (72 %) patients without findings on EUA [17, 19, 26]. Although a total of 60 primaries were identified in the lingual tonsils, the location was specified for only 49 patients [15, 17–19, 26, 27]. Of these 49 tumors, 46 (94 %) were identified in the ipsilateral base of tongue and 3 (6 %) were found in the contralateral base of tongue.

**Table 6:** Identification rate of lingual tonsillectomy in the presences of other findings

Author	Physical Exam		DI (CT/MRI)		PET/CT		PE/DI/PET-CT		EUA with biopsy	
	+	-	+	-	+	-	+	-	+	-
Abuzeid et al. [26]	1/1 (100 %)	0/0 (0 %)	1/1 (100 %)	0/0 (0 %)	1/1 (100 %)	0/0 (0 %)	1/1 (100 %)	0/0 (0 %)	0/0 (0 %)	1/1 (100 %)
Blanco et al. [28]	-	-	-	-	-	-	-	-	-	-
Durmus et al. [18]	0/0 (0 %)	4/14 (29 %)	0/0 (0 %)	4/14 (29 %)	-	-	-	-	-	-
Karni et al. [16]	0/0 (0 %)	11/18 (61 %)	0/0 (0 %)	11/18 (61 %)	-	-	-	-	-	-
Mehta et al. [19]	0/0 (0 %)	9/10 (90 %)	0/0 (0 %)	9/10 (90 %)	4/4 (100 %)	5/6 (83 %)	4/4 (100 %)	5/6 (83 %)	0/0 (0 %)	9/10 (90 %)
Mourad et al. [27]	0/0 (0 %)	1/1 (100 %)	-	-	1/1 (100 %)	0/0 (0 %)	1/1 (100 %)	0/0 (0 %)	1/1 (100 %)	0/0 (0 %)
Nagel et al. [17]	0/0 (0 %)	13/19 (68 %)	-	-	-	-	-	-	-	8/14 (57 %)
Patel et al. [15]	-	-	-	-	-	-	13/25 (52 %)	8/16 (50 %)	-	-
**Total**	**1/1 (100 %)**	**38/62 (61 %)**	**1/1 (100 %)**	**24/42 (57 %)**	**6/6 (100 %)**	**5/6 (83 %)**	**19/31 (61 %)**	**13/22 (59 %)**	**1/1 (100 %)**	**18/25 (72 %)**

*Abbreviations*: *DI* diagnostic imaging, *CT* computed tomography, *MRI* magnetic resonance imaging, *PE* physical examination, *PET* positron emission tomography, *EUA* panendoscopic examination under anesthesia

### Adverse events for TORS/TLM

Table 7 shows the adverse events reported in studies of TORS or TLM. In total, six studies [15, 17–19, 26, 27] reported overall perioperative complication rates for 117 patients and all eight studies [15–19, 26–28] reported perioperative mortality rates for 139 patients. Additionally, four studies [15, 17, 19, 28] reported hemorrhage rates for 97 patients, one study [18] reported tracheostomy rates for 22 patients, three studies [18, 19, 28] reported gastrostomy rates for 36 patients, and five studies [17–19, 26, 28] commented on return to diet for 73 patients.

**Table 7:** Adverse events following TORS/TLM

Author	Hemorrhage	Tracheostomy	Gastrostomy	No Return to diet	Other	Deaths	Total Complications
Abuzeid et al. [26]	-	-	-	0/1 (0 %)	-	0/1 (0 %)	0/1 (0 %)
Blanco et al. [28]	0/4 (0 %)	-	0/4 (0 %)	0/4 (0 %)	0/4 (0 %)^a^	0/4 (0 %)	-
Durmus et al. [18]		0/22 (0 %)	0/22 (0 %)	0/22 (0 %)	-	0/22 (0 %)	0/22 (0 %)
Karni et al. [16]	-	-	-	-	-	0/18 (0 %)	-
Mehta et al. [19]	0/10 (0 %)	-	1/10 (10 %)^b^	1/10 (10 %)^b^	-	0/10 (0 %)	2/10 (20 %)
Mourad et al. [27]	-	-	-	-	-	0/1 (0 %)	0/1 (%)
Nagel et al. [17]	1/36 (3 %)^c^	-	-	0/36 (0 %)	0/36 (0 %)	0/36 (0 %)	1/36 (3 %)
Patel et al. [15]	4/47 (9 %)^d^	-	-	-	1/47 (2 %)^e^	0/47 (0 %)	5/47 (11 %)
**Total**	**5/97 (5 %)**	**0/22 (0 %)**	**1/36 (3 %)**	**1/73 (1 %)**	**1/87 (1 %)**	**0/139 (0 %)**	**8/117 (7 %)**

^a^No patients developed esophageal strictures

^b^Patient was a heavy smoker (60 packs/year) with an identified HPV-negative 2.0 cm submucosal tongue base tumor

^c^Postoperative tonsil bleed requiring return to OR

^d^Two patients required return to OR

^e^One patient had tongue swelling requiring one additional day of observation before discharge

The most common complication was hemorrhage in 5 of 97 (5 %) patients, of which three (3 %) required return to the operating room for hemostasis. None (0 %) of the 22 patients with available outcomes required tracheostomy, and only 1 of 36 (3 %) patients required a gastrostomy tube. In this single patient, the requirement for a permanent gastrostomy tube was due to adjuvant chemoradiation and heavy tobacco use in the post-operative period [19]. Furthermore, only 1 of 73 (1 %) patients did not tolerate return to diet within 24 h post-operatively. Other perioperative complications such as tongue swelling [15] occurred in 1 of 87 (1 %) patients. There were no perioperative deaths resulting from TORS or TLM.

## Discussion

Localization of the primary tumor in patients with cervical metastasis of unknown origin remains a challenging yet important goal. When available diagnostic modalities fail to detect a primary tumor, treatment typically consists of large volume radiation to the neck as well as potential primary mucosal sites with or without chemotherapy, or neck dissection with or without adjuvant chemoradiation [2–7]. Head and neck irradiation may be associated with dysphagia, xerostomia, mucosal atrophy, and osteoradionecrosis of the jaw [11, 29, 30]. Identification of the primary tumor site may mitigate these risks by minimizing radiotherapy volumes and also allowing for more directed radiation, potentially sparing the pharyngeal constrictors, salivary glands, and mandible. Furthermore, depending on the margin status and pathological features of the primary tumor identified (and resected) by any of these approaches, one may elect to avoid radiotherapy to mucosal surfaces and manage the neck disease in isolation. The implications of this strategy warrant further study.

The goal of this systematic review was to determine the effectiveness of TORS and TLM in localizing an occult primary tumor and to elucidate the role of these techniques within the traditional diagnostic paradigm. Our findings demonstrated that TORS/TLM can increase the detection of occult primary tumors at all stages of the diagnostic workup. We also aimed to determine the incremental benefit of using these techniques by analyzing the identification rate of unknown primaries in a subgroup of patients undergoing lingual tonsillectomies. Many of the patients who are managed with TORS and TLM undergo a palatine tonsillectomy in addition to lingual tonsillectomy. While a palatine tonsillectomy can be performed using more cost-effective traditional approaches, a lingual tonsillectomy, on the other hand, may require the superior visualization and exposure afforded by these techniques. In the present study, the identification rate of a primary tumor using lingual tonsillectomy was 60/108 (56 %).

Currently, there is no standard diagnostic algorithm for an unknown primary tumor. The typical workup includes physical examination and diagnostic imaging consisting of CT and/or MRI. The addition of PET and PET/CT have resulted in improved detection rates ranging from 15 to 28 % [1, 4, 9, 11, 31] and 32 to 44 % [4, 8, 32], respectively. Studies have also reported successful primary tumor identification using PET/CT in the presence of unremarkable findings on physical examination, imaging, and panendoscopy, with identification rates ranging from 28 to 37 % [4, 33, 34]. However, PET and PET/CT does not reliably detect tumors smaller than 8 to 10 mm in diameter [35]. Interestingly, in our study of 111 identified primary tumors, we reported an average tumor diameter of 1.15 cm, with 57 % of primary tumors less than 10 mm in diameter. This finding may suggest that many of the primary tumors in this setting are below the detection level of PET-CT imaging. Another limitation of PET imaging is the high false-positive rate due to physiologic uptake in the lymphoid tissue of Waldeyer’s ring. Recent reviews have reported false-positive rates as high as 39 % for PET and 37 % for PET/CT [8, 9]. These false positives may (incorrectly) guide treating physicians to target treatment volumes based on the areas of uptake. Histopathologic corroboration with tissue is needed prior to making treatment decisions.

Surgical evaluation of an unknown primary involves the use of examination under anesthesia with biopsies of clinically and radiologically suspicious sites. Studies also show that palatine tonsillectomy improves the detection rate compared to tonsil biopsy in patients with demonstrable tonsillar tissue [12, 14]. Overall, a comprehensive diagnostic workup including physical examination, imaging, and panendoscopy with directed biopsies and/or tonsillectomy reveals a primary tumor site in 19 to 53 % of patients [1, 3–5, 10, 36]. However, in the absence of remarkable physical examination or radiological findings, detection rates are only 17 to 29 % [5, 10, 36].

In comparison, our present study demonstrates significantly higher identification rates using TORS/TLM compared to traditional diagnostic techniques, suggesting that evaluation of the lingual tonsil with the aid of TORS/TLM has clinical benefit in the work-up of unknown primary tumors. In contrast to the detection rates reported above, our review of the literature revealed an identification rate of 79 % in the presence of remarkable findings on physical examination and imaging, and a 92 % detection rate in the presence of remarkable findings on panendoscopy. Most importantly, the detection rate remained high at 71 % in the absence of findings on physical examination and imaging (including PET/CT), and 67 % even after failed EUA with directed biopsies. This highlights a potential role for TORS/TLM in the diagnostic algorithm of these patients as a “final step” after failed panendoscopy.

Similar findings were noted in the subgroup of patients undergoing lingual tonsillectomy using TORS or TLM. The detection rate was 61 % among patients with remarkable findings on physical examination and radiological imaging, and remained at 59 % among patients with unremarkable findings. Furthermore, lingual tonsillectomy was successful in identifying the primary tumor in 18 of 25 (72 %) patients even after failed EUA with biopsies. These data also support the use of TORS and TLM to perform a lingual tonsillectomy as a “last resort” when all other diagnostic modalities have failed to localize a primary tumor site.

Some authors advocate for upfront lingual tonsillectomy in the initial management of occult primary tumors rather than awaiting the results of directed biopsies of the pharynx [16, 17]. This approach may reduce the delay to diagnosis and definitive treatment, and also obviate the need for a second operation in the event of positive biopsy results. Our data showed that lingual tonsillectomy identified the primary tumor site in 60 of 108 patients (56 %) overall, supporting a potential role for upfront lingual tonsillectomy in select patients with unknown primary tumors. Disadvantages of this approach include a longer initial operation, and exposure to potentially unnecessary surgery and associated risks of perioperative complications. Our review of the literature revealed that the complication rate of TORS/TLM, while relatively low (7 %), was not zero [15, 17–19, 26]. The potential impact on quality of life (QOL) is another important consideration, with a recent study demonstrating a significant decline in multiple QOL domains such as speech, eating, aesthetics, and social disruption up to 12 months post-treatment with TORS [20]. Further research is needed to evaluate long-term QOL outcomes following TORS/TLM and investigate the role of lingual tonsillectomy in the initial work up of occult primary tumors.

Our findings corroborate previous studies that suggest that subsites of the oropharynx such as the palatine tonsil and tongue base are the most common sites of occult primary tumors [5, 10, 12, 15]. This is likely due to the fact that small primary tumors can be hidden in areas that are difficult to visualize such as the palatine and lingual tonsillar crypts. The justification for performing a palatine tonsillectomy for detection of a hidden primary tonsillar cancer can similarly be applied to the tongue base, where a lingual tonsillectomy is necessary to identify small hidden primaries.

The issue of “bilaterality” or contralateral tumor resection is one that warrants discussion. In our study, we report a contralateral primary tumor in the tongue base in 6 % of the contralateral tongue base and 15 % in the contralateral tonsil. This proportion is comparable to previous reports which have found bilateral or contralateral palatine tonsil disease in 10 to 23 % of identified primary tumors [13, 37, 38]. However, it remains unclear whether these represent multiple primary tumors or multicentric disease that has been described in human papillomavirus (HPV) mediated oropharyngeal carcinoma compared to isolated contralateral disease [24, 39, 40]. Regardless, these findings support the use of bilateral palatine and/or lingual tonsillectomy as part of a comprehensive diagnostic workup or staged resection of the contralateral palatine tonsil and lingual tonsil in the event that no primary is found on the ipsilateral side. However, clinical judgment is required to weigh potential benefits and risks, and determine the optimal approach for each individual patient [13–16].

This study is limited by the small sample size of included studies, particularly for the subgroup of patients receiving lingual tonsillectomy, as well as the heterogeneity between diagnostic workup performed at different institutions. This is not surprising given the relatively recent advent of this expanded surgical paradigm in the investigation of head and neck carcinoma of unknown primary. Our study highlights the need for a standardized diagnostic and treatment approach to unknown primary tumors that also considers emerging transoral surgical procedures such as TORS and TLM. Inter-institutional and inter-surgeon variation in the technique used to perform lingual tonsillectomy could have also affected our findings, particularly given that only three studies [17, 19, 26] provided a general description of this procedure. More frequent and detailed reporting on surgical technique is needed to further investigate the impact of inter-institutional variation on identification rates. Another potential limitation is publication bias, as institutions with more favorable results may be more likely to publish their findings, particularly for the newer TORS/TLM techniques. Despite these limitations, this is the first systematic review to comprehensively evaluate the use of TORS/TLM and lingual tonsillectomy specifically for the identification of primary head and neck squamous cell carcinoma with an unknown primary site. By pooling data from multiple institutions with varying methods of preoperative assessment, we were able to gather a relatively large sample size and minimize single-surgeon and single-institution biases. Future prospective studies with more patients and standardized diagnostic and treatment protocols are needed to further investigate the usefulness and cost-effectiveness of these newer transoral surgical techniques, and corroborate the encouraging results presented in this study.

## Conclusion

This systematic review supports the use of TORS and TLM to aid in the identification of a primary head and neck squamous cell carcinoma of unknown origin, with superior detection rates compared to the traditional diagnostic workup. We also demonstrate that the addition of formal lingual tonsillectomy using TORS/TLM is a safe and effective option that can increase the yield of localizing an occult primary tumor. Identification of the primary tumor using minimally-invasive transoral techniques reduces treatment-induced morbidity and permits directed management, thereby potentially improving survival and functional outcomes.

## Abbreviations

CT: computed tomography
DI: diagnostic imaging
EUA: examination under anesthesia
HPV: human papillomavirus
MRI: magnetic resonance imaging
PE: physical examination
PET: positron-emission tomography
QOL: quality of life.
TLM: transoral laser microsurgery
TORS: transoral robotic surgery
